# Time perspective and psychological hardiness mediate a COVID-19 related life threat

**DOI:** 10.1192/j.eurpsy.2021.1223

**Published:** 2021-08-13

**Authors:** E. Nikolaev

**Affiliations:** Social And Clinical Psychology, Ulianov Chuvash State University, Chebokasry, Russian Federation

**Keywords:** time perspective, psychological hardiness, life threat, COVID-19

## Abstract

**Introduction:**

Unpredictable risks of COVID-19 morbidity and mortality make people suffer from threats and fears. Are there any psychological personality traits that correlate with a decrement in such feelings?

**Objectives:**

To specify the relationship between psychological characteristics of time perspective, hardiness and COVID-19-related life threat.

**Methods:**

We used Zimbardo Time Perspective Inventory, Maddi Hardiness Scale and a 17-point Attitude towards COVID-19 Questionnaire to question 327 Russian university students on social networks. The survey was carried out in the second half of May 2020 in the period of increasing COVID-19 morbidity and mortality rates. The mean age of the respondents was 21.24±2.84; most of them were females (61.16%).

**Results:**

The survey showed that every fifth respondent had a high level of COVID-19-related life threat (21.10 %). At the same time, every tenth of the respondents (10.09%) saw no threat to their life in the situation of a fast spreading dangerous novel coronavirus infection. According to the correlation analysis, decrement in feeling personal threat related to the spread of COVID-19 was directly associated (p<0.05) with low indicators on the scales of Negative Past (r=0.16), Hedonistic Present (r=0.13) and Fatalistic Present (r=0.17). Certain inverse relation was found between the level of COVID-19 related life threat and such indicators as psychological hardiness – commitment (r=-0.16), and challenge (r=-0.23).

**Conclusions:**

Dispositional orientation to the present and future, as well as psychological characteristics of hardiness may mediate COVID-19 related life threat; therefore, these may be used as a possible basis for preventing stress and mental disorders in population.

